# Factors influencing fatigue in UK nurses working in respiratory clinical areas during the second wave of the Covid‐19 pandemic: An online survey

**DOI:** 10.1111/jocn.16375

**Published:** 2022-05-25

**Authors:** Nicola J. Roberts, Kareena McAloney‐Kocaman, Kate Lippiett, Emma Ray, Lindsay Welch, Carol A. Kelly

**Affiliations:** ^1^ School of Health and Life Sciences Glasgow Caledonian University Glasgow UK; ^2^ School of Health Sciences University of Southampton Southampton UK; ^3^ Respiratory Research Centre Edge Hill University Ormskirk UK

**Keywords:** anxiety, COVID‐19, depression, fatigue, nursing, respiratory

## Abstract

**Aims and objectives:**

This study explores UK nurses' experiences of working in a respiratory clinical area during the COVID‐19 pandemic over winter 2020.

**Background:**

During the first wave of the pandemic, nurses working in respiratory clinical areas experienced significant levels of anxiety and depression. As the pandemic has progressed, levels of fatigue in nurses have not been assessed.

**Methods:**

A cross‐sectional e‐survey was distributed via professional respiratory societies and social media. The survey included Generalised Anxiety Disorder Assessment (GAD7), Patient Health Questionnaire (PHQ9, depression), a resilience scale (RS‐14) and Chalder mental and physical fatigue tools. The STROBE checklist was followed as guidance to write the manuscript.

**Results:**

Despite reporting anxiety and depression, few nurses reported having time off work with stress, most were maintaining training and felt prepared for COVID challenges in their current role. Nurses reported concerns over safety and patient feedback was both positive and negative. A quarter of respondents reported wanting to leave nursing. Nurses experiencing greater physical fatigue reported higher levels of anxiety and depression.

**Conclusions:**

Nurses working in respiratory clinical areas were closely involved in caring for COVID‐19 patients. Nurses continued to experience similar levels of anxiety and depression to those found in the first wave and reported symptoms of fatigue (physical and mental). A significant proportion of respondents reported considering leaving nursing. Retention of nurses is vital to ensure the safe functioning of already overstretched health services. Nurses would benefit from regular mental health check‐ups to ensure they are fit to practice and receive the support they need to work effectively.

**Relevance to clinical practice:**

A high proportion of nurses working in respiratory clinical areas have been identified as experiencing fatigue in addition to continued levels of anxiety, depression over winter 2020. Interventions need to be implemented to help provide mental health support and improve workplace conditions to minimise PTSD and burnout.

1 **Impact Statement**

Nurses are vital for health systems and their mental and physical wellbeing need to be nurtured and protected.

Nurses and possibly other healthcare professionals are likely to have fatigue levels higher than the general population and it is vital that they are supported to provide safe and effective care for patients. Appropriate support and interventions need to be implemented to improve the mental health and provide support for staff working in clinical areas.

2 **What does this paper contribute to the wider global clinical community?**

- Although this study is UK‐based, its findings resonate with other international studies, emphasising the global importance of understanding the long‐term effect of pandemics on fatigue in healthcare professionals and its impact on anxiety and depression.
- It is essential that the global clinical community considers the impact of pandemics on the mental health and wellbeing of healthcare professionals and puts in place robust systems to support them.
- Our study found a large proportion of nurses were considering leaving the profession. Global healthcare employers should be alert to the potential impact on retention of experienced nurses and should take proactive measures to recruit, retain and upskill the workforce.

## 1 INTRODUCTION

Worldwide, there have already been more than 256 million cases of COVID‐19 with mortality rates of over 5 million (World Health Organisation, 2021). The ongoing pandemic has driven the NHS to make adaptations to the delivery of care (Wanat et al., 2021). The unrelenting workload has been mentally and physically challenging for many healthcare professionals, particularly those based in critical care and respiratory services, with increased morbidity and mortality in these professional groups (Marsh, 2020). In the United Kingdom, Shaw et al. reported feelings of hopelessness and helplessness amongst healthcare professionals working in the National Health Service (UK) (Shaw, 2020). Our previous work has shown that a significant proportion of nurses working in respiratory clinical areas experienced high levels of anxiety and/or depression during the first wave of the pandemic last March (Roberts, Kelly, et al., 2021; Roberts et al., 2021). We previously highlighted the inconsistencies in provision of work‐based mental health support and the impact of the pandemic on nurses' lives both at home and at work during the first wave of the pandemic (Roberts, Kelly, et al., 2021; Roberts, McAloney‐Kocaman, et al., 2021). Building on our previous work, this study identifies and characterises the experiences of nurses working in respiratory clinical areas in terms of anxiety, depression, resilience and fatigue over the winter of 2020. Our body of work aimed to inform policy decision‐making in supporting the healthcare professional workforce through the current pandemic, in preparation for future pandemics and to make recommendations for future respiratory workforce planning.

## 2 BACKGROUND

The COVID‐19 outbreak has forced many nurses and other healthcare professionals globally to function under extreme pressure with, at some points, limited or inadequate resources. Nurses have had to adapt quickly to demanding situations, including frequent changes to protocols and procedures and staff shortages, as well as the demands of delivering advanced and palliative care to very sick patients. Despite this, nurses have reported an increased sense of duty, dedication to patient care and personal sacrifice (Fernandez et al., 2020).

The impact of this has meant that many nurses and healthcare professionals have experienced mental health issues including anxiety, depression and, in some cases, even post‐traumatic stress disorder (PTSD) (Pfefferbaum and North, 2020). Moore et al. have shown that nearly two‐thirds of frontline healthcare professionals working with COVID‐19 reported feeling anxious during April 2020 (Moore and Kolencik, 2020). Sagherian et al. (2020) found that during the first wave of the pandemic (May –June 2020), nurses experienced poor sleep, fatigue and multiple psychological problems. Furthermore, staff sickness levels significantly increased, rising above the 10‐year average and continued to rise during the second wave (September 2020–January 2021) peaking at 2,231,693 lost days (FTE), of which 28.6% were COVID‐19‐related (NHS Digital., 2021).

Several studies have explored the prevalence of ‘burnout’ in healthcare professionals during the COVID‐19 pandemic. Burnout can be both a process and an outcome of challenging work conditions that causes physical and emotion exhaustion, alienation from work‐related activities and poor performance (Maslach and Leiter, 2016; Dall'Ora et al., 2020). Dall'Ora et al. (2020) provide seven key areas impacting the likelihood of burnout: work life, workload and staffing levels, job control, reward and fairness, shift‐work and working patterns, psychological demands and job complexity, support factors (relationships/leadership) and work environment and hospital characteristics (Dall'Ora et al., 2020). Denning et al. (2021) found that 67% of healthcare professionals screened positively for burnout during the first wave of the pandemic. A systematic review and meta‐analysis demonstrated nurses, in particular, experienced high levels of burnout during this period (Galanis et al., 2021). This study examines the levels of fatigue in nurses which can be used as an indicator (or precursor) to potential burnout.

Longitudinal data from previous epidemics indicate that the psychological impact on healthcare professionals may be long lasting with increased risk of burnout, depression, anxiety, substance misuse and post‐traumatic stress disorder (PTSD) (Hou et al., 2020;Wu et al., 2008). As the COVID‐19 pandemic continues, it is important to identify and address the short‐, medium‐ and long‐term effects it may have on nurses and other healthcare professionals.

This study, therefore, aimed to identify and characterise the experiences of nurses working within respiratory areas during mid‐November 2020 to the end of January 2021, specifically focusing on fatigue, and exploring the relationship between fatigue and anxiety, depression and resilience. It is important to understand the impact of working during a prolonged pandemic on nurses' mental health and to address how they can be better supported. First, because healthcare providers have a professional and moral responsibility to maintain a safe working environment for their staff. Second, to avoid experienced clinical staff leaving the profession which can leave significant gaps in service provision (Gellasch, 2015).

## 3 AIMS

This study explored levels of fatigue in the nursing population working in respiratory clinical areas during the second wave in order to investigate what variables, if any, impact on fatigue levels.

## 4 METHODS

### 4.1 Design of the survey tool

The survey tool consisted of 91 questions using a mixture of open‐ended and closed questions, including a Likert scale (0 no confidence to 10 highest confidence). Participants could also add free‐text comments. The survey included the following four validated tools: the Chalder fatigue tool (Chalder et al., 1993), the Generalised Anxiety Disorder Assessment (GAD7, a commonly used tool for screening for generalised anxiety disorder) (Spitzer et al., 2006), the Patient Health Questionnaire (PHQ9, depression scale) (Spitzer et al., 1999) and the resilience scale RS‐14 (Wagnild, 2009). The STrengthening the Reporting of OBservational studies in Epidemiology (STROBE) statement (von Elm et al., 2008) (Appendix S1) was followed when producing the manuscript.

Data were collected on demographic characteristics such as age, gender, ethnicity, number of years qualified, geographical location, nursing background and clinical setting and role. We asked nurses about their views on changes to the delivery of patient care and their clinical role during the pandemic.

The GAD7 score was scored as minimal anxiety (0–4), mild anxiety (5–9), moderate anxiety (10–15) and severe anxiety (15–21). Depression using the PHQ9 scale was scored as none to minimal depression (0–4), mild depression (4–9), moderate depression (10–14), moderately severe depression (15–19) and severe depression (20–27). The Chalder Fatigue Scale was developed for hospital and community studies of patients with chronic fatigue syndrome (Wessely and Powell, 1989) and has been previously used to determine levels of fatigue in nurses during COVID‐19 (Zhan et al., 2020). The tool consists of 11 items measuring fatigue‐related symptoms in the following two domains: physical and mental. Those without fatigue would score 11/33, indicating that they had fatigue ‘no more than usual’, studies in health communities score between 12–14 (Cella and Chalder, 2010; Loge et al., 1998). For resilience scores, low resilience is found with scores below 65; scores between 65–81 illustrate moderate resilience; scores above 81 indicate high levels of resilience (Wagnild and Young, 1993; Wagnild, 2009).

We piloted the survey tools with a small group of nurses from the teams' professional network for construct validation and to estimate completion time and guard against overburden to this already pressured group of nurses. We made minor changes to enhance ease of understanding.

The online survey used RedCAP© and was analysed in SPSS (Version 25.0). We cascaded the link to the survey via social media (Twitter, LinkedIn, Facebook). Professional respiratory societies (British Thoracic Society, Primary Care Respiratory Society, Association for Respiratory Nurse Specialists) were also asked to circulate the survey link via email and social media. We redistributed the survey link regularly over a 10‐week period from mid‐November 2020 to the end of January 2021.

### 4.2 Sampling method

We did not undertake a sample size calculation as we were unsure of the likely response rate given the uncertainties of winter pressures on the NHS, combined with a rise in COVID‐19 infection rates and consequent healthcare utilisation. We do not know the number of respiratory nurses in the UK; however, our previous survey received a response rate of 255 at the start of the pandemic. With the added pressures and long‐term impact of the pandemic this number would be unrealistic. Thus, we proposed a minimum convenience sample of approximately 150 participants: UK registered adult nurses working in respiratory clinical areas from mid‐November 2020 to the end of January 2021.

### 4.3 Data analysis

Survey data was entered into SPSS^©^ (Version 25.0) for analysis. Descriptive statistical analysis and univariate inferential testing (Mann–Whitney, Kruskal–Wallis, level of significance *p* < .05) were undertaken for the survey response to explore relationships with the respective dependent scores for Chalder fatigue, GAD‐7, PHQ‐9 and for the purpose of variable reduction in regression modelling.

The study team undertook a series of multiple logistic regression models to provide an indication of the relative independent association of the socio‐demographic variables, COVID‐19‐related experiences, working conditions and mental health (depression, anxiety and resilience) with the outcome variables (Chalder fatigue, physical and mental). We collapsed variable categories for the regression analysis, to reduce the number of independent variables, and entered these into separate multiple linear regression models for each dependent variable. Only independent variables significantly associated with the outcomes in the univariate analysis were entered into the regression models. Multicollinearity was assessed using correlation analysis, and no independent variables were removed on this basis.

### 4.4 Ethics statement

The survey included participant information at the start of the survey tool; consent was inferred from completion of survey. We signposted mental health follow‐up in case any participants became inadvertently distressed by answering questions. We anonymised all data collected, removing any identifiable information prior to analysis. The School of Health and Life Sciences committee at Glasgow Caledonian University (HLS/NCH/19/036) approved the study.

## 5 RESULTS

We received 161 responses for the survey, predominately from women (86%), aged over 35 (82.6%). In total, 95% (153/161) of respondents classed themselves as white; only a small sample of other ethnic groups completed the survey. Respondents were from all countries in the UK, with the majority from England (66%, 107/161). Just under 63% (101/161) usually worked in an acute setting. Most participants were Agenda for Change Band 7 (Advanced Nurse/Nurse Practitioner) or above (58.9%, 95/161) and were more than 20 years post qualified (52.2%) (Table 1). Nearly, 17% (27/161) reported having had COVID‐19; an additional 12.4% (20/161) suspected having had COVID‐19 but did not have the infection confirmed as testing was not then available.

**TABLE 1:** Demographic characteristics

	Frequency (%)
Age (years) (*n* = 161)	
Under 35	28 (17.3)
Over 35	133 (82.6)
Years qualified (*n* = 161)	
0–20	77 (47.8)
20+	84 (52.2)
NHS Band (*n* = 161)	
5/6	66 (41.0)
7/8	95 (58.9)
Have you had time off work because of work related stress during the pandemic (*n* = 161)	
Yes	8 (5.0)
No	153 (95.0)
Were you able to keep up to date with training requirements during the pandemic (*n* = 161)	
Yes	83 (51.6)
No	78 (48.4)
Do you feel that there has been changes to patient safety compared to prior to the pandemic? (*n* = 161)	
Yes	119 (73.9)
No/Not sure	42 (26.1)
Is the quality of care you give the same as pre‐pandemic? (*n* = 161)	
Yes	80 (49.7)
No	81 (50.3)
Do you think patients are satisfied with the care they have received?	
Yes	87 (54.0)
No/Not sure	74 (45.9)
Are there plans to redeploy staff in your organisation	
Yes	82 (50.9)
No/Not aware yet	79 (49.1)
Do you feel adequately prepared for your current clinical role if there is another peak of COVID cases? (mean, SD)	7.30 (2.13)
Have you considered leaving nursing because of the pandemic? (Yes)	41 (25.5)
Have you had the COVID‐19 infection? (*n* = 161)	
Yes	27 (16.8)
No	114 (70.8)
Unconfirmed – testing not available	20 (12.4)
Have your career prospects been affected by the pandemic	
Yes – positively	26 (16.0)
Yes‐ negatively	26 (16.0)
No – no change	113 (70.1)
Have you experienced any emotional responses from patients since the start of the pandemic (*n* = 161)	
Yes – positive	139 (85.8)
Yes – Negative	113 (69.8)
Resilience (*n* = 158)	
Very low	6 (3.8)
Low	4 (2.5)
On the low end	21 (13.3)
Moderate	42 (26.6)
Moderately high	60 (38.0)
High	25 (15.8)
Mean score (SD)	80.7 (11.67)
Median (Min, Max)	82.0 (21, 98)
Anxiety (*n* = 159)	
Minimal anxiety (0–4)	83 (52.2)
Mild anxiety (5–9)	40 (25.2)
Moderately severe anxiety (10–14)	17 (10.7)
Severe anxiety (15–21)	19 (12.0)
Mean Score (SD)	6.16 (5.63)
Median (Min, Max)	4.0 (0, 21)
Depression (*n* = 160)	
Minimal depression (0–4)	79 (49.4)
Mild depression (5–9)	47 (29.4)
Moderate depression (10–14)	20 (12.5)
Moderately severe depression (15–19)	10 (6.3)
Severe depression (20–27)	4 (2.5)
Mean Score (SD)	5.87 (5.32)
Median (Min, Max)	5.0 (0, 26)
Chalder fatigue score (Likert) (*n* = 156)	
Physical fatigue (Qu 1–7)	10.29 (4.04)
Mental fatigue (Qu 8–11)	5.54 (2.44)
Mean Score (SD)	15.87 (5.94)
Median (Min, Max)	15.0 (0, 32)

### 5.1 Fatigue, anxiety, depression and resilience scores

The mean overall Chalder fatigue score was 15.87 ± 5.94, with a score of 10.29 ± 4.04 for *physical fatigue* and 5.54 ± 2.44 for *mental fatigue*. Those without fatigue would score 11/33, indicating that they had fatigue ‘no more than usual’. The total fatigue scores were higher than those found in community studies and studies investigating the general population (Loge et al., 1998; Cella and Chalder, 2010). The median score for anxiety (GAD‐7) was 4 (range 0–21), representing minimal anxiety (0–4 minimal, 5–9 mild, 10–15 moderate and 15–21 severe anxiety). Frequencies show that most nurses, 51.6% (83/161) experienced minimal anxiety, 24.8% (40/159) experienced mild anxiety and 22.4% (36/161) experienced moderate severe to severe anxiety (Table 1). Scores were similar for depression, median scores were 5 (range 0, 26), indicating mild depression (0–4 none to minimal, 4–9 mild, 10–14 moderate, 15–19 moderately severe and 20–27 severe depression). The largest proportion (49.0%, 79/161), experienced none to minimal depression symptoms, 29.2% (47/161) had mild symptoms and 21.1% (34/161) experiencing moderate to severe symptoms (Table 1). The median score for resilience was 80.7 (range 21, 98), only 19.6% (31/158) had resilience at the low end of the scale and below, 80.3% (127/158) had a moderate or moderately high resilience score. Other studies have shown median scores of between 70–72 for young adolescents (median 71 [min 14, max 97] *n* = 400), young adolescents with special needs (median 73 [min 17, max, 98] *n* = 656) and students (median 73 [min 14, max 98] *n* = 1659) (Surzykiewicz et al., 2019). Higher resilience was significantly associated with lower anxiety (χ ^2^ *p* = .002) and lower depression scores (χ ^2^ *p* < .001). Lower anxiety scores were significantly associated with lower depression scores (χ ^2^ *p* < .001). As can be seen in Table 2, higher median Chalder fatigue scores for both domains and total scores were associated with lower resilience (all *p* < .01) and higher levels (>10) of both anxiety and depression (all *p* < .005).

**TABLE 2:** Univariate analysis

		Chalder physical (*n* = 157)		Chalder mental (*n* = 159)		Chalder total fatigue score (*n* = 156)	
		Median (min, max)	Sig	Median (min, max)	Sig	Median (min, max)	Sig
Age	Under 35	13.0 (0, 21)	**.005**	7.0 (0, 11)	**.004**	20.5 (1, 32)	**.001**
	35+	10.0 (0, 21)		5.0 (0, 11)		14.0 (0, 32)	
Years Qualified	Up to 20 years	11.0 (0, 21)	**.046**	6.0 (0, 11)	.316	17.0 (0, 32)	**.045**
	>20 years	9.0 (0, 21)		5.(0, 11)		14.0 (0, 32)	
Time off work due to stress	Yes	13.5 (7, 21)	**.009**	7.0 (4, 11)	.064	21.0 (11, 32)	**.016**
	No	10.0 (0, 21)		5.0 (0, 11)		15.0 (0, 32)	
Able to keep up with training requirements	Yes	9.0 (0, 18)	**.003**	5.0 (0, 11)	**.007**	14.0 (0, 27)	**.001**
	No	11.0 (1, 21)		6.0 (1, 11)		18.0 (3, 32)	
Has there been changes to patient safety compared to pre‐pandemic	Yes	10.0 (0, 21)	.183	5.0 (0, 11)	.072	16.0 (0, 32)	.068
	No/Not sure	9.0 (0, 20)		4.0 (0, 11)		14.0 (0, 29)	
Have you experienced any positive emotional responses from patients	Yes	10.0 (0, 21)	.512	5.0 (0, 11)	.062	15.0 (0, 32)	.212
	No	11.0 (0, 21)		6.0 (0, 11)		17.0 (0, 31)	
Have you experienced any negative emotional responses from patients	Yes	9.5 (0, 21)	.165	5.0 (0, 11)	.713	15.0 (0, 32)	.534
	No	11.0 (0, 17)		5.0 (0, 11)		15.0 (1, 26)	
Have you had the COVID infection	Yes	10.0 (0, 21)	.207	6.0 (0, 11)	.390	17.0 (0, 32)	.301
	No	10.0 (0, 21)		5.0 (0, 11)		14.0 (0, 32)	
	Unconfirmed	10.5 (0, 21)		5.0 (1, 10)		16.0 (1, 31)	
Have you considered leaving nursing because of the pandemic	Yes	12.0 (0, 21)	**.000**	7.0 (0, 11)	**.000**	18.0 (0, 32)	**.000**
	No	9.0 (0, 20)		4.5 (0, 11)		14.0 (0, 29)	
Do you feel prepared for your role if there is another peak Scale 1–10	1–5	11.0 (5, 20)	.185	6.0 (3, 11)	**.048**	17.0 (8, 29)	.107
	6–10	10.0 (0, 21)		5.0 (0, 11)		14.0 (0, 32)	
Have your career been affected (positively) by the pandemic	Yes	11.0 (1, 21)	.289	7.0 (2, 11)	**.029**	18.0 (3, 32)	.100
	No	10.0 (0, 21)		5.0 (0, 11)		15.0 (0, 32)	
Resilience score	Very low – on the low end	13.0 (6, 21)	**.002**	7.0 (2, 11)	.004	19.0 (11, 32)	**.001**
	Moderate – High	10.0 (0, 21)		5.0 (0, 11)		14.0 (0, 32)	
Anxiety score	0–9	9.0 (0, 20)	**.000**	5.0 (0, 11)	**.000**	14.0 (0, 29)	**.000**
	10–21	13.5 (6, 21)		8.0 (3, 11)		21.0 (9, 32)	
Depression score	0–9	9.0 (0, 20)	**.000**	4.0 (0, 11)	**.000**	14.0 (0, 29)	**.000**
	10–27	15.0 (9, 21)		8.0 (2,11)		22.0 (12, 32)	

Mann–Whitney, Kruskal–Wallis tests undertaken, level of significance *p* < .05. Significant values are highlighted in bold.

### 5.2 Regression analysis

Several variables were identified as potentially significantly important (Table 2) in influencing fatigue scores including age, years qualified, time off work, keeping up with training, changes to patient safety, leaving nursing, preparedness for next wave, positive career changes, resilience, anxiety and depression scores. Each of these was entered into a multiple linear regression with the outcome fatigue measures. The sample size was appropriate for multiple linear regression analysis [12] The results of the multiple linear regression models are shown in Table 3. All significant variables (cut‐off *p* < .05) were entered into linear regression models for fatigue (physical and mental domains and total score).

**TABLE 3:** Regression for Chalder physical, mental and total fatigue

Variable	Model 1		Model 2		Model 3	
	Chalder physical		Chalder mental		Total Chalder fatigue	
	β	*p*	β	*p*	β	*p*
Age						
18–34 (ref)						
35 and older	.057	.410	.018	.810	.041	.548
Time off work due to stress						
No (ref)						
Yes	.064	.340			.039	.556
Able to keep up with training requirements						
No (ref)						
Yes	**−.134**	**.041**	−.138	.056	**−.160**	**.014**
Have you considered leaving nursing because of the pandemic						
No (Ref)						
Yes	.056	.417	.122	.098	.087	.207
Do you feel prepared for your role if there is another peak			−.102	.163		
Have your career been affected (positively) by the pandemic						
No (Ref)						
Yes			**.161**	**.026**		
Anxiety						
Mild (ref)						
Moderate – high	**.158**	**.043**	.141	.091	**.172**	**.026**
Depression						
Mild (ref)						
Moderate – high	**.457**	**<.001**	**.293**	**.001**	**.428**	**<.001**
Resilience						
Low (ref)						
High	−.057	.406			−.073	.287
*R* ^2^	.42		.31		.43	

Significant values are highlighted in bold.

#### 5.2.1 Model 1 Chalder physical fatigue score

As can be seen in Table 3, the independent variables explained 42% of the variance in physical fatigue. Lower levels of physical fatigue were significantly associated with being able to keep up with training requirements (*p* < .05), and higher level of physical fatigue associated with levels of anxiety and depression above the recommended thresholds (*p* < .05 and *p* < .001, respectively).

#### 5.2.2 Model 2 Chalder mental fatigue score

The independent variables explained 31% of the variance in mental fatigue. Surprisingly, mental fatigue scores were significantly higher among those who felt their career had been positively impacted by the pandemic (*p* < .05), and, less surprisingly, by those who scored above the recommended threshold for depression (*p* < .001).

#### 5.2.3 Model 3 Chalder total fatigue score

Finally, the independent variables explained 43% of the variance in total fatigue score. Lower levels of total fatigue were significantly associated with being able to keep up with training requirements (*p* < .05), while higher total fatigue was associated with levels of anxiety above the recommended threshold (*p* < .05), and levels of depression above the recommended threshold (*p* < .001).

### 5.3 Professional development

A small proportion of respondents reported that they had time off work due to work‐related stress (5%, 8/161) during the pandemic; most were able to keep up with training requirements during the pandemic (51.6%, 93/161). Most felt reasonably confident about being adequately prepared for another peak of COVID‐19 cases (mean score 7.30 ± 2.13; 1 = not adequately prepared; 10 = completely prepared). A small proportion of respondents felt that the pandemic had positively affected their career prospects (16%, 26/161); most felt there had been no change (70.1% 113/161). Just over a quarter (25.5%, 41/161) reported that they had considered leaving nursing because of the pandemic.

### 5.4 Clinical care, safety and risk

Most stated that they had experienced positive emotional interactions from patients since the start of the pandemic (85.8%, 139/161). Examples of positive emotional responses such as:

In the 1st lock down, lots of encouraging messages from patients, thankful we remained open.

[R28]

Nurses also reported negative emotional responses from patients (69.8% 113/161) for example:

‘Anger, frustration and fear and anxiety all relating to concerns re covid’ and ‘Anger that you don't have staffing levels to meet their needs’.

[R18]

The majority of participants thought that patient safety had been negatively affected by the pandemic (73.9%, 119/161):

Barriers in providing same level of patient care due to patient isolation and increased PPE use required.

[R8]

All areas are affected but increased pressure resulting in pressured decisions which I believe impact patient care.

[R111]

Due to staff and ward movements, I feel skill mix has been inadequate at times in regards to patient safety.

[R34]

Just over half thought the quality of care did not meet the same standards as pre‐pandemic levels (50.3%, 81/161):

Clinical basics yes but trying to achieve excellent care has been difficult.

Conditions hard, PPE difficult to work in. Fewer nurses lots of sickness. Workforce tired psychologically exhausted. Still trying hard.

[R40]

There was one exception where a nurse commented that the pandemic may, paradoxically, have improved care:

All staff are going above and beyond. There has not been a lack of care, if anything there has been improvements to the patient's care with new MDT ways of working.

[R93]

Just over half (54%, 87/161) thought patients were satisfied with the care they had received, one respondent wrote:

I think there will be arguments for both! I think people do see how hard we're working under very difficult and strenuous circumstances but there will be people that will be unsatisfied with the care we are able to give.

[R150]

Just over 50% (50.9%, 82/161) reported that there were plans to redeploy staff in their organisation over the winter period.

## 6 DISCUSSION

This study set out to identify and characterise the experiences of nurses working in respiratory settings, specifically focusing fatigue and the influence of anxiety, depression and resilience over winter 2020. Our previous study showed significant levels of clinically relevant anxiety and depression in nurses working in respiratory clinical areas during the first wave of the pandemic (Roberts, Kelly, et al., 2021; Roberts, McAloney‐Kocaman, et al., 2021). In this study, anxiety scores were broadly the same as our previous study (moderate–severe anxiety 21% [40/191, 1st survey] versus 22.8% [36/158, 2nd survey]). Depression scores were slightly higher (moderate–severe depression 17.2% [31/181, 1st survey] versus 21.3% [34/160, 2nd survey]). This is slightly lower than the reported 33% (*n* = 2600) of respondents reported experiencing severe or extremely severe depression, anxiety or stress in a recent survey of UK nurses and midwives' concerns during the COVID‐19 pandemic study (Mitchell, 2020).

For healthcare professionals, psychological distress caused by work pressure, resulting in anxiety and depression, is not unexpected. Data from the UK during the pandemic have shown an increase in the level of anxiety and depression in the general population overall, compared with previous years, due to uncertainty, isolation and lack of access to mental health services (Jia et al., 2020). A large survey of healthcare professionals across eight European countries found higher levels of clinically significant anxiety and depression among nurses and doctors, during the pandemic particularly in the UK and France, compared with participants from non‐medical occupations (Hummel et al., 2021). If psychological distress persists, in the form of anxiety and depression, this may lead to long‐term effects, poor resilience levels, fatigue and burnout resulting in an increase in sickness levels and effects to overall health which would impact finite NHS resources (Elliott, 2017; Ghassemi, 2021). For the health service, this could be detrimental in terms of safe staffing levels and long‐term recruitment and retention as, whilst there has been a surge in applications to study nursing, experienced and highly qualified nurses may leave the NHS sooner than they had planned because of burnout (RCN, 2021). In this study, worryingly a quarter of respondents were considering leaving the NHS and most nurses expressed concerns over the quality of clinical care and safety; this is not unique to the UK (Gellasch, 2015). Being able to maintain standards, with effective leadership, good teamwork and adequate resources is an important aspect for job satisfaction in nursing and, if not present, leads to nurses feeling demoralised and dissatisfied (Senek et al., 2020).

Despite reported anxiety and depression, few nurses in the current study took time off work with stress and their resilience scores remained moderate to high in both our surveys (moderate to high 146/180, 81.1%; 127/158, 80.4%). Overall median resilience scores were moderate (score 82, min‐14, max‐98) meaning individuals may possess some of the characteristics of resilience but this need strengthening (Wagnild, 2009). The authors of the scale identified five common components of resilience: equanimity, perseverance, self‐reliance, meaningfulness and existential aloneness ((Wagnild and Young, 1993; Wagnild, 2011). This may be because most of the participants in our sample were from higher clinical bands and were, therefore, more experienced and possibly had better resilience, very few participants felt that the pandemic had benefitted their career prospects.

Higher resilience was also significantly associated with lower anxiety (Chi square *p* = .002) and lower depression scores (χ ^2^ *p* < .001). A systematic review of resilience levels in healthcare workers across the world during the COVID‐19 pandemic found that nurses had similar levels of resilience in their analysis, although in the USA resilience levels dropped, whilst in China they increased (Baskin et al., 2021). Differences may be due to governmental approaches in managing the spread of COVID‐19, the structure of healthcare systems as well as nurse training and education(Duncan, 2020). Our findings that higher levels of resilience are associated with lower levels of psychological distress reflect that of other studies of healthcare workers during the pandemic (Bozdağ and Ergün, 2020). Restubog and colleagues discuss the mental impact of emotional regulation strategies to manage negativity: interacting with family/peers who make you feel good, undertaking things you enjoy and keeping busy(Restubog et al., 2020). Bozdağ and Ergün (2020) found that protective factors for resilience in healthcare workers during the pandemic were good sleep, positive emotions and life satisfaction (Bozdağ and Ergün, 2020). Adequate personal resilience can act as a protective factor against more serious long‐term psychological disturbance and is, therefore, an important characteristic to possess for nurses working in the NHS during the COVID‐19 pandemic (Wesley and Powell, 1989). (Bozdağ and Ergün, 2020).

In this study, we found significant levels of physical and mental fatigue in respondents (mean physical fatigue score 10.29 [4.04]; mental fatigue score 5.54 [2.44], total fatigue 15.87 [5.94]). This is higher than a general population study (Loge et al., 1998) which had total fatigue scores for women of 12.6, and 11.9 for men but comparable to the study looking at fatigue after COVID infection in the general population (Townsend et al., 2020).

The results from the regression analyses show that high physical fatigue is associated with not being able to keep up with training requirements. Higher anxiety and depression scores were also associated with high physical fatigue, depression was also significantly associated with greater mental fatigue. For the total fatigue score, being able to complete training needs was protective; however, high levels of anxiety or depression were associated with higher scores for total fatigue. Teng et al. also found a correlation between fatigue, anxiety and depression (Teng et al., 2020). Fatigue and other symptoms are strong predictors of depression (Corfield et al., 2016).

### 6.1 Strengths and limitations

This study was conducted during the second wave of the pandemic in the UK, during a period where nurses were being surveyed by several organisations and research groups about their experiences during the pandemic. Respondents may have been fatigued by taking part in numerous surveys, explaining the lower recruitment numbers for this study. We sought a convenience sample and recruited through social media platforms and via professional networks for ease, but we may have excluded relevant nursing groups if they were not working because of sickness, had left the profession, were not a member of a professional society or did not use social media. This study did have similar demographics to the previous study, which may indicate some of the same people took part as we used the same recruitment methods, but the survey was anonymous and unmatched. Both surveys had a high female/Caucasian presence, as well as older, more experienced and nurses with a higher job grading and, therefore, may not be as representative as warranted, although this does reflect the demographics of UK respiratory nurses in other studies (Yorke et al., 2017).

## 7 CONCLUSIONS

Nurses make up the highest proportion of staff in the UK National Health service, nurse retention is essential to the smooth running of healthcare systems. Employers, including the NHS, need to be more proactive rather than reactive in addressing the mental and physical needs of staff who have been under increased work‐related pressure since the start of the pandemic.

Just over a quarter of respondents reported considering leaving nursing. To retain nurses, attract staff and reduce sickness levels (which have been high during the pandemic), nurses would benefit from regular mental health check‐ups to ensure they are fit to practice and receive the support and care they need to function safely and work effectively. This could involve regular use of assessment tools such as the stress risk assessment tool (ASSET), often used to manage those with existing health conditions in the workplace (Faragher et al., 2004). Resources have been put in place during the pandemic, such as drop‐in sessions and telephone support, this needs to continue and be built on. Nurses identified as needing further mental health support could be offered one to one or group sessions to address their specific needs. This could be in the form of counselling, group sessions on resilience or referral to their GP. Future research should assess strategies to improve resilience levels and retention of nurses. Feedback mechanisms, from staff and patients, to report safety concerns, need to be strengthened; furthermore, the NHS needs to be responsive to this feedback. NHS staff have performed extremely well under difficult circumstances, but where improvements can be made these need to be highlighted and actioned upon.

The repercussions of the pandemic are likely to last for many years and there is concern over future epidemics. We need to support, train and nurture healthcare staff to ensure they stay in their professional roles, which in turn will maintain standards and benefit patients and society. Recruitment and retention of nursing staff needs to be a national and international priority.

## 8 RELEVANCE TO CLINICAL PRACTICE

A high proportion of nurses experienced anxiety and depression during the 2nd wave of COVID in winter 2020. Nurses also experienced higher total fatigue scores compared with the general public. Higher anxiety and depression scores were associated with high physical fatigue. Effective interventions need to be implemented into the NHS to help improve provide mental health support and improve conditions in the workplace to reduce the potential for mental ill health and burnout from the impact of the pandemic. These interventions need to be available for all and be tailored to individuals to fit into workplans and shift patterns and provide protected time and support to improve staff mental health and wellbeing.

## 9 AUTHOR CONTRIBUTIONS

Nicola Roberts: conceptualization, methodology, formal analysis, investigation, writing – original draft, project administration. Kareena McAloney‐Kocaman: methodology, formal analysis, writing – reviewing and editing. Kate Lippiett: conceptualization, methodology, investigation, writing– reviewing and editing. Emma Ray: conceptualization, methodology, investigation, writing – reviewing and editing. Lindsay Welch: conceptualization, methodology, investigation, writing– reviewing and editing. Carol Kelly: conceptualization, methodology, investigation, writing– reviewing and editing.

## 10 CONFLICT OF INTEREST

No conflict of interests declared.

11

## Supporting information


Appendix S1

## Data Availability

Research data are not shared.
